# Association between prevalence and severity of chronic kidney disease and employment status: a nationwide study in Korea

**DOI:** 10.1186/s12889-023-17338-4

**Published:** 2024-01-18

**Authors:** Seoyeong Choi, Suk-Yong Jang, Eunjeong Choi, Yu shin Park

**Affiliations:** 1 Department of Public Health, Graduate School, Yonsei University, 50 Yonsei-ro, Seodaemun-gu, Seoul, 03722 Republic of Korea; 2 Institute of Health Services Research, Yonsei University, 50 Yonsei-ro, Seodaemun-gu, Seoul, 03722 Republic of Korea; 3 Department of Healthcare Management, Graduate School of Public Health, Yonsei University, 50 Yonsei-ro, Seodaemun-gu, Seoul, 03722 Republic of Korea

**Keywords:** Chronic kidney insufficiency, Chronic disease, Employment, Glomerular filtration rate

## Abstract

**Background:**

The prevalence of chronic kidney disease (CKD) is increasing globally, and understanding the association between CKD and employment status is crucial. This cross-sectional study aimed to investigate the association of CKD with employment and occupation type among patients with CKD.

**Methods:**

We analyzed data from 36,732 Korean adults aged ≥ 30 years, who participated in the Korean National Health and Nutrition Examination Survey between 2014 and 2021. CKD was detected based on the estimated glomerular filtration rate, and the employment status of the participants was classified into distinct categories: full-time permanent employment, unemployment, self-employment, and precarious employment. We analyzed the data using multiple logistic regression.

**Results:**

We observed a significant association between CKD and a higher likelihood of unemployment compared to that in individuals without CKD (odds ratio, 1.70; 95% confidence interval, 1.47–1.96). This association was more prominent in patients with severe CKD. In the multivariable logistic analysis, patients with CKD had a higher likelihood for precarious employment (odds ratio, 1.29; 95% confidence interval, 0.92–1.88), self-employment (odds ratio, 1.3; 95% confidence interval, 0.90–1.88), and unemployment (odds ratio, 2.10; 95% confidence interval, 1.51–2.92) compared to individuals without CKD.

**Conclusions:**

Our study demonstrated that CKD is associated with a higher likelihood of unemployment and engagement in precarious employment. These findings highlight the challenges faced by patients with CKD in obtaining stable employment and emphasize the need for interventions to improve the employment outcomes of individuals with CKD.

## Introduction

The increasing prevalence of chronic kidney disease (CKD) worldwide and its impact on the quality of life of individuals with CKD are closely associated with the burgeoning elderly population [1]. Between 1990 and 2017, there was a notable increase of 29.3% in the global prevalence of CKD, and the worldwide prevalence of CKD in 2017 was estimated to be 9.1% (95% UI 8.5 to 9.8) [2]. Moreover, the burden of CKD has increased substantially in terms of its incidence, prevalence, death, and disability-adjusted life years (DALYs) [2, 3]. In Korea, the proportion of individuals aged 65 years is projected to reach approximately 40% by 2050, positioning the country as the fastest-aging nation among The Organization for Economic Cooperation and Development (OECD) countries [4]. In 2021, the prevalence of CKD in Korea was approximately 8.4%, and over the past decade, the number of patients with CKD receiving medical treatment and healthcare expenses has doubled: it was 110,000 in 2011 and was estimated to increase to 280,000 in 2021 [5]. In particular, healthcare expenses have increased from 1 billion dollars to 2.2 billion, making CKD the most financially burdensome disease per patient in Korea [5].

Work serves as a means for most individuals to meet their essential life needs, offering opportunities for personal achievement, self-realization, and cultivation of self-esteem [6]. Extensive evidence supports a strong association between unemployment and adverse health outcomes, including heightened anxiety and depression, diminished self-esteem, and deteriorated physical well-being [6]. Several previous studies have reported that chronic diseases can affect patients’ work participation. People with chronic diseases are less likely to be employed, work fewer hours than average [7], and face a diminished quality of life compared to those without such conditions [8].

CKD encompasses a range of disorders that affect kidney structure and function [9]. Owing to reduced functional capacity and endurance, patients with CKD encounter limitations in their daily activities [10]. They endure health-related issues, including physical symptoms, reduced work capacity, and psychological distress, as well as environmental challenges, such as prolonged waiting times for nephrology care and the inconvenience of frequent hospital visits [11]. Loss of kidney function and work-related problems can negatively affect work opportunities among patients with CKD. A qualitative study revealed that patients with CKD frequently experience obstacles in sustaining employment, financial difficulties, and the stigma associated with the illness [12]. Furthermore, high unemployment rates among patients undergoing dialysis or transplant have been documented in various countries, such as the United States [13], England [14], Netherlands [15] indicating a global phenomenon. Demographic and socioeconomic disadvantages faced by patients with CKD can contribute to their heightened vulnerability to unemployment [16].

Previous studies have predominantly examined the impact of employment status on the development of chronic diseases, such as cancer, stroke, and diabetes [17–19]. The risk factors associated with the health outcomes related to CKD have been investigated [20, 21]; however, there remains a dearth of knowledge regarding the relationship between the prevalence of CKD and its socioeconomic consequences, particularly with regard to employment. Although substantial progress has been made in policy discussions on other chronic diseases [22], there is a notable scarcity of research on CKD.

Prior investigations pertaining to CKD and unemployment have primarily focused on patients undergoing dialysis or kidney transplant, who represent the group with severe CKD grades, and compared unemployment rates among patients undergoing hemodialysis, peritoneal dialysis, and kidney transplantation [13–15, 23]. In contrast, our study aimed to compare the unemployment rates between individuals with and without CKD. Additionally, we stratified patients with CKD based on CKD severity to investigate variations in unemployment rates based on disease severity. We also examined the types of occupations among patients with CKD and their trends.

Furthermore, the previous studies had limited sample sizes, included only patients with CKD without a comparison group, and primarily focused on Western populations [16, 23]. In contrast, our study used data from the Korean National Health and Nutrition Examination Survey (KNHANES), a representative survey encompassing the entire Korean population, to investigate the association between CKD and employment, making it the first of its kind in East Asia.

## Methods

### Data

We utilized KNHANES data from 2014 to 2021 in this study. The KNHANES is an annual nationwide cross-sectional survey conducted by the Korea Centers for Disease Control and Prevention. This comprehensive survey comprises three distinct components: a health interview, health examination, and nutrition survey. The health interview and examination are conducted through face-to-face to capture information pertaining to socioeconomic status, health behaviors, biochemical profiles (including fasting blood serum and urine samples), food intake, and dietary behaviors [24]. Demographic and social characteristics were obtained through the health interviews, and trained medical personnel collected health examination data, including blood pressure and laboratory data. The KNHANES employs a complex, stratified, multistage clustered probability design to ensure a nationally representative sample of the South Korean population.

### Study participants

**Fig. 1:**
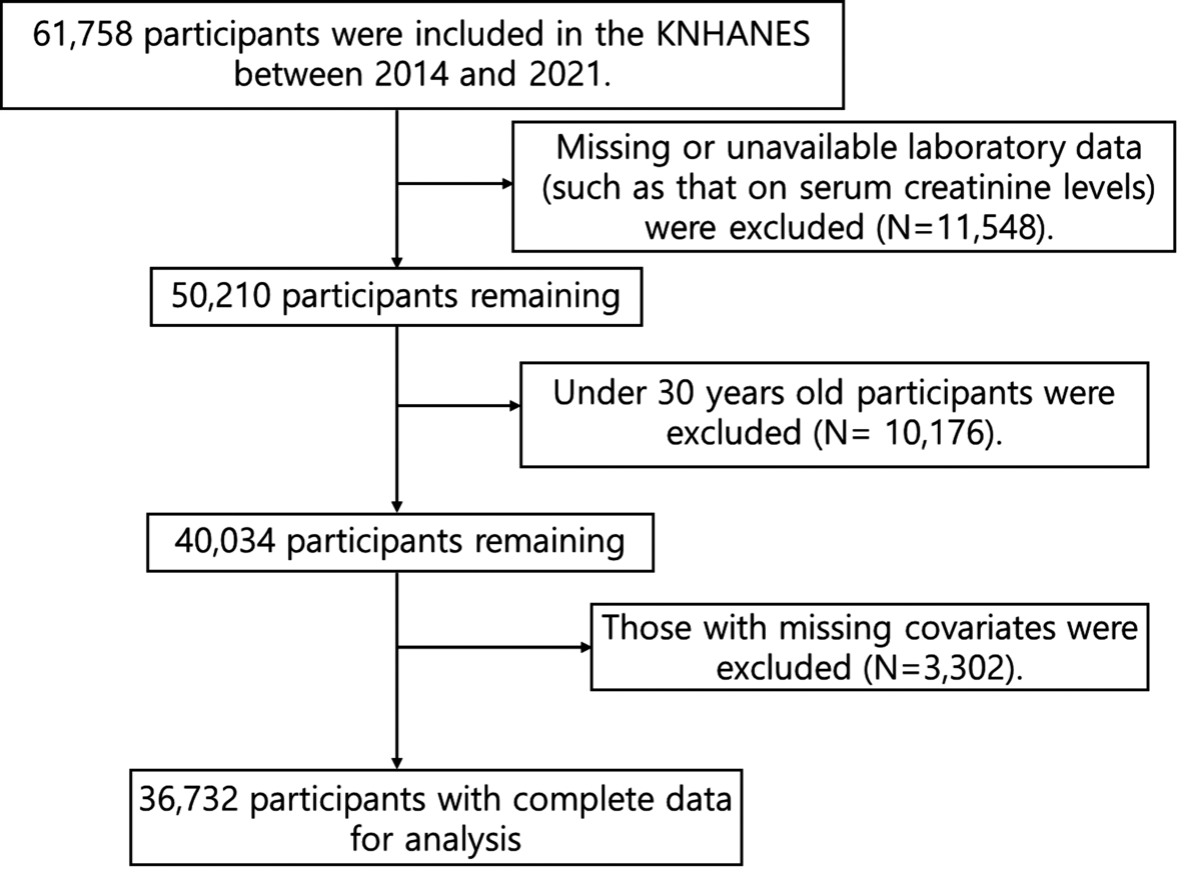
Flow diagram for study sample

In total, 61,758 participants were included in the KNHANES between 2014 and 2021. Participants those with missing or unavailable laboratory data (such as that on serum creatinine levels, HbA1c) (N = 11,548), aged under 30 years old (N = 10,176), and those with missing covariates (N = 3,302) were excluded from the analysis. The final analysis included 36,732 participants (Fig. 1. Flow diagram for study sample).

### Variables

#### CKD status

The main variable of interest (independent variable) was the CKD status. The Modification of Diet in Renal Disease (MDRD) equation is widely recognized as an accurate formula for estimating glomerular filtration rate (GFR) [25]. As serum creatinine levels were measured in the KNHANES, we calculated the estimated glomerular filtration rate (eGFR) in this study using the MDRD formula: 175 ^− 1.154^ × age ^− 0.203^ (× 0.742 if female) [26]. Serum creatinine levels were measured in fasting blood samples using the kinetic Jaffe rate reaction method. Participants with an eGFR of < 60 mL/min/1.73 m² were assigned to the CKD group [27].

Moreover, CKD was categorized based on the Kidney Disease Improving Global Outcomes (KDIGO) guidelines [28], indicating the presence of moderate-to-severe impairment in renal function. For sensitivity analyses, we additionally classified the participants into three distinct eGFR categories: ≥60, 45 to < 60, and < 45 mL/min/1.73 m², as well as ≥ 60, 30 to < 60, and < 30 mL/min/1.73 m², aligning with preserved kidney function, mild-to-moderate renal decline, and moderate-to-severe kidney dysfunction, respectively, following the KDIGO classification system [29].

#### Assessment of employment status

Based on employment status, the participants were classified as employed (coded as “1”) or unemployed (coded as “0”). Furthermore, employment status was divided into four distinct categories of work status: full-time permanent, self-employed, precarious, and unemployed. The classification of precarious employment was based on the Korean Welfare Panel Study, which provides distinct categorization of employment status in South Korea. Consistent with previous research, we adopted the same definitions for full-time permanent employment, unemployment, and precarious employment [30]. Specifically, individuals who met all four of the following criteria were classified as having a permanent job: (i) direct employment by the employer (not subcontracted or dispatched workers or self-employed without employees), (ii) full-time work (not part-time), (iii) absence of a fixed-term employment contract (not temporary workers), and (iv) high job security with a low likelihood of job loss (not day laborers) [30]. Precarious employment was defined as not meeting all the four criteria. Participants who were not employed at the time of the survey were classified as unemployed. Self-employed individuals are defined as those who operate their own businesses with a distinct employment status [30].

#### Other covariates

To account for the potential confounding variables associated with the independent and dependent variables, the analysis included the following covariates selected based on previous studies: demographic variables (age, sex, education level, and marital status), socioeconomic variables (household income quartile), and health-related variables (diabetes, hypertension, alcohol consumption, smoking, physical activity, and obesity) [21, 31]. All selected covariates were categorized according to the study objectives, with categories of age groups (30–39, 40–49, 50–59, 60–69, and > 70 years), education level (below elementary school, middle school, high school, or college and above), marital status, and household income quartiles.

Comorbidities, such as diabetes mellitus (DM) and hypertension, were included as variables that strongly influenced CKD and employment [32, 33] DM was diagnosed based on glycated hemoglobin (HbA1c) levels > 6.5% [34]. DM was categorized into four groups: normal, prediabetic, controlled (HbA1c < 6.5%), and uncontrolled (HbA1c > 6.5%). Hypertension was classified as normal, prehypertension, controlled (systolic blood pressure [SBP] < 140 and diastolic blood pressure [DBP] < 90), and uncontrolled (SBP ≥ 140 and DBP ≥ 90). Smoking status was self-reported, and participants were categorized as never, former, or current smokers. Problem drinking was defined as consuming six or more units of alcohol at least twice per week for men and four or more units of alcohol at least twice per week for women. Physical activity was defined as engaging in a minimum of 150 min of moderate-intensity physical activity, 75 min of vigorous-intensity physical activity, or an equivalent combination of physical activity per week [35]. The participants were classified into three groups based on body mass index (BMI) – underweight (BMI < 18.5), normal weight (18.5 ≤ BMI < 25), and obese (BMI ≥ 25) – in accordance with the standards established by the Korean Society for the Study of Obesity [36].

#### Statistical analysis

This study applied a complex sampling design that included multistage clustering and stratification to represent the South Korean population based on a complex sample provided by the KNHANES. The data analysis was performed in three parts. First, a chi-square test was performed to assess the distribution of general characteristics in the CKD and non-CKD groups. Second, multivariable-adjusted logistic regression was performed to investigate the relationship between CKD and employment status after adjusting for covariates. Third, a subgroup analysis was conducted to compare the employment status of participants based on odds ratios (OR) and 95% confidence intervals (CI) to determine the strength of the association. In addition, multinomial logistic regression was performed to examine the association between CKD prevalence and employment status, which was further categorized into four types of job status. Proc Survey procedures, including the weight, strata, and cluster statements of SAS (version 9.4 M6; SAS Institute Inc., Cary, NC, USA), were used for all statistical analyses.

## Results

Table 1 presents the general characteristics of the study population according to the CKD status. The study included 36,732 individuals, of whom 1,722 (4.7%) were diagnosed with CKD and 35,010 (95.3%) comprised the non-CKD group. Compared to the non-CKD group, patients with CKD exhibited characteristics of older age (61.5%), higher proportion of males (51.9%), lower educational attainment (49.7%), and lower household income levels (44.9%). A higher proportion of patients with CKD exhibited uncontrolled comorbidities, such as DM (26.6%) and hypertension (31.4%), than the non-CKD group. Additionally, a significant majority of patients with CKD engaged in uncontrolled drinking (92.9%).

**Table 1:** General characteristics of the study population

Variables	Chronic kidney disease^a^						
	TOTAL		Yes		No		*P*
	N	%	N	%	N	%	
	36,732	100	1,722	4.7	35,010	95.3	
**Age**							< 0.0001
30–39	6341	17.2	15	0.9	6326	18.1	
40–49	7650	20.8	59	3.4	7591	21.7	
50–59	8168	22.2	154	8.9	8014	22.9	
60–69	7707	20.9	435	25.3	7272	20.8	
70 ≤	6866	18.6	1059	61.5	5807	16.6	
**Sex**							< 0.0001
Male	15,982	43.4	894	51.9	15,088	43.1	
Female	20,750	56.3	828	48.1	19,922	56.9	
**Diabetes** **mellitus (HbA1c, %)** ^**b**^							< 0.0001
Normal	18,248	49.5	492	28.6	17,756	50.7	
Pre-Diabetes	12,137	32.9	523	30.4	11,614	33.2	
Controlled	2320	6.3	249	14.5	2071	5.9	
Not controlled	4027	10.9	458	26.6	3569	10.2	
**Hypertension**							< 0.0001
Normal	14,547	39.5	217	12.6	14,330	40.9	
Pre-Hypertension	9027	24.5	216	12.5	8811	25.2	
Controlled	6624	18	748	43.4	5876	16.8	
Not controlled	6534	17.7	541	31.4	5993	17.1	
**Marriage status**							< 0.0001
Yes	34,077	92.5	1685	97.9	32,392	92.5	
No	2655	7.2	37	2.1	2618	7.5	
**Education** ^**c**^							< 0.0001
Under Elementary school	8121	22	855	49.7	7266	20.8	
Middle school	4190	11.4	236	13.7	3954	11.3	
High school	11,130	30.2	380	22.1	10,750	30.7	
College and over	13,291	36.1	251	14.6	13,040	37.2	
**Smoking status**							< 0.0001
Current	6300	17.1	226	13.1	6074	17.3	
Former	8585	23.3	551	32	8034	22.9	
Never	21,847	59.3	945	54.9	20,902	59.7	
**Physical activity** ^**d**^							< 0.0001
Yes	21,273	57.7	1227	71.3	20,046	57.3	
No	15,459	42	495	28.7	14,964	42.7	
**Problem drinking** ^**e**^							< 0.0001
Yes	30,814	83.6	1600	92.9	29,214	83.4	
No	5918	16.1	122	7.1	5796	16.6	
**Obesity (BMI, kg/m2)**							< 0.0001
Underweight (< 18.5)	1136	3.1	35	2	1101	3.1	
Normal (18.5 ≤ BMI < 25)	22,283	60.5	919	53.4	21,364	61	
Obese (≥ 25)	13,313	36.1	768	44.6	12,545	35.8	
**Household income**							< 0.0001
Low	6979	18.9	773	44.9	6206	17.7	
Mid-low	9113	24.7	434	25.2	8679	24.8	
Mid-high	10,070	27.3	273	15.9	9797	28	
High	10,570	28.7	242	14.1	10,328	29.5	

Abbreviations: CKD, chronic kidney disease; DM, diabetes mellitus; HbA1c, hemoglobin A1c; BMI, body mass index

Values with unweighted frequency (weighted %) are presented

^a^ chronic kidney disease defined as a result of MDRD study equations less than 60mL/min/1.73m^2^

^b^ 6.5% of HbA1c is standard for the evaluation and management of DM, Korean Diabetes Association

^c^ Education is classified as current academic background. dropout, enrollment, and absence are classified as previous academic background

^d^ Physical activity is defined as an aerobic physical activity for a week, Korea Disease Control and Prevention Agency

^e^ Problem drinking is defined as 6 units of alcohol two or more times per week in men and 4 units of alcohol two or more times per week in women

**Fig. 2:**
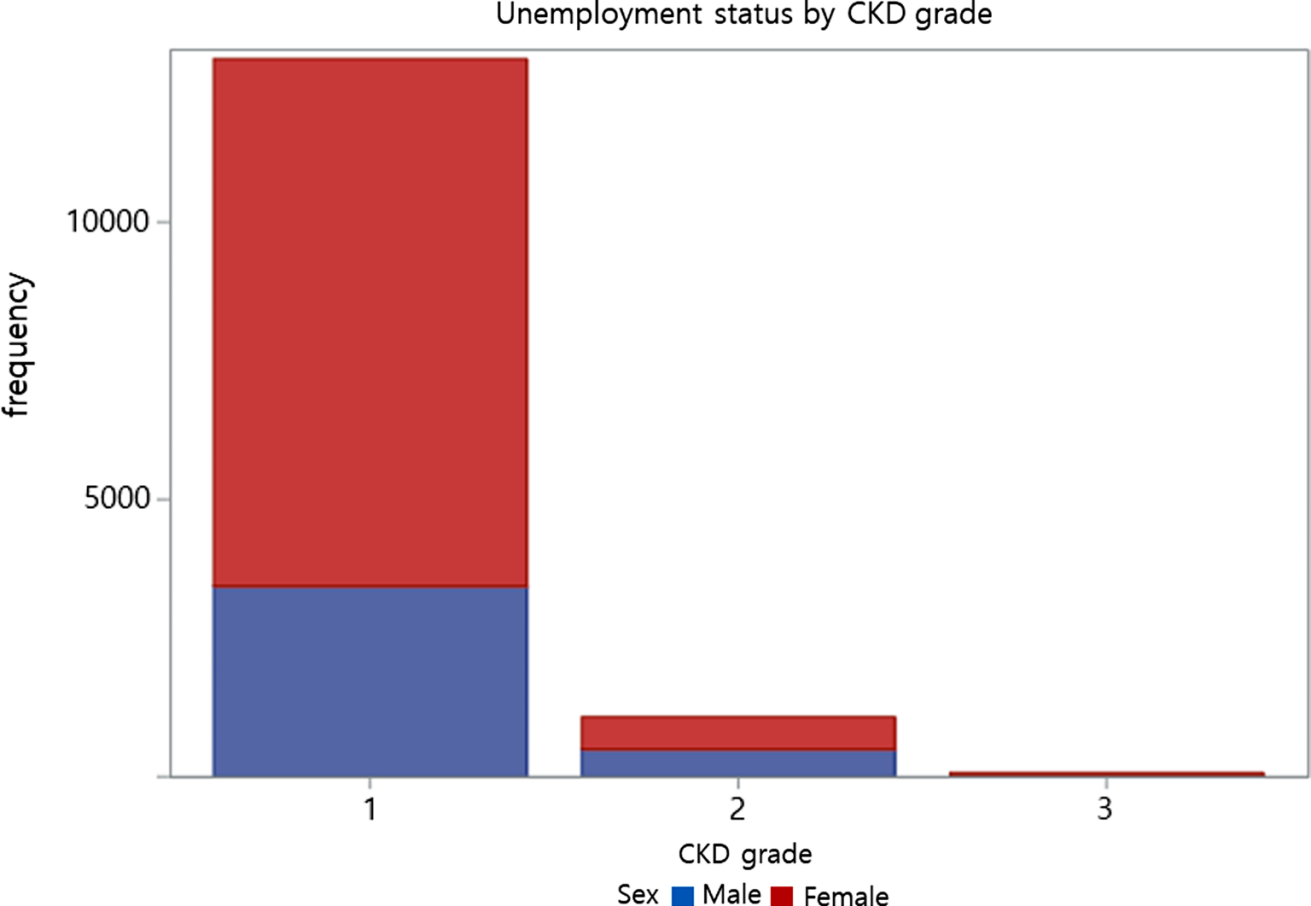
Bar chart for unemployment status by CKD grade. The CKD grade shown in the figure is classified according to the estimated glomerular filtration rates (eGFR). CKD grade 1 indicates that an eGFR of 60 or higher was classified as non-CKD, and eGFRs of 30 to 60 and less than 30 mL/min/1.73m^2^ were classified as grades 2 and 3

Tables 2, 3, 4 and 5 were adjusted for potential confounding covariates; age, sex, marriage status, education status, household income, physical activity, smoking and drinking status, obesity, hypertension, diabetes mellitus. The associations among CKD prevalence, severity, and work status are presented in Table 2; Fig. 2. Table 2 presents several models to convey the differences in CKD severity and kidney function (eGFR) while adjusting for potential confounding covariates. In Model 1, CKD patients were 1.70 times more likely to be unemployed compared to individuals without CKD (OR, 1.70; 95% CI, 1.47 to 1.96). This trend persisted across Models 2, 3, and 4, indicating a higher likelihood of unemployment with an increase in CKD severity; statistically significant associations were observed.

**Table 2:** Association between prevalence and severity of CKD and work status

CKD	Status of unemployment (N = 14,104)						
	N	%	OR		95% CI		*p for trend*
**Model 1**							-
No	12,948	91.8	1				
Yes	1,156	8.9	1.7	(1.47	–	1.96)	
**Model 2** ^**a**^							< 0.001
No (≥ 60)	12,948	91.8	1				
45–60	1,081	7.7	1.61	(1.37	–	1.89)	
< 45	75	0.6	2.05	(1.53	–	2.76)	
**Model 3** ^**b**^							< 0.001
No (≥ 60)	12,948	91.8	1				
30–60	862	6.1	1.68	(1.45	–	1.94)	
< 30	294	2.1	2.1	(1.11	–	3.96)	
**Model 4** ^**c**^							< 0.001
No (≥ 60)	12,948	91.8	1				
45–60	862	6.1	1.61	(1.37	–	1.88)	
30–45	219	1.6	2.03	(1.45	–	2.84)	
15–30	54	0.4	1.42	(0.66	–	3.05)	
< 15	21	1.8	5.05	(2.23	–	11.43)	

Abbreviations: CKD, chronic kidney disease; OR, odd ratio; CI, confidence interval

Values with unweighted frequency (weighted %) are presented

“status of unemployment” was adjusted for age, sex, marriage status, education status, household income, physical activity, smoking and drinking status, obesity, hypertension, diabetes mellitus

^a^ Estimated glomerular filtration rates of 60 or higher was classified as non-CKD, 45 to 60 and less than 45 mL/min/1.73m^2^ were classified as grades 1 and 2, respectively

^b^ Estimated glomerular filtration rates of 60 or higher was classified as non-CKD, 30 to 60 and less than 30 mL/min/1.73m^2^ were classified as grades 1 and 2, respectively

^c^ Estimated glomerular filtration rates of 60 or higher was classified as non-CKD, 45 to 60, 30 to 45, 15 to 30 and less than 15 mL/min/1.73m^2^ were classified as grades 1 through 4, respectively

For each of the trait analyzed, the OR’s were estimated from a single logistic model that included age, sex, marriage status, education status, household income, physical activity, smoking and drinking status, obesity, hypertension, diabetes mellitus as covariates

**Table 3:** Association of CKD prevalence with unemployment stratified by each level of covariates

Variables	CKD			
	NO		YES	
		**OR** ^*^	**95% CI**	***p for interaction***
**Sex**				< 0.0001
Male	1	1.67	(1.37–2.04)	
Female	1	1.52	(1.24–1.86)	
**Age**				0.985
60 or over	1	1.53	(1.28–1.83)	
30 ~ 59	1	2.16	(1.75–2.66)	
**Household income**				0.472
High (3,4 quartile)	1	1.72	(1.45–2.04)	
Low (1, 2 quartile)	1	1.56	(1.20–2.03)	
**Education**				0.803
Under high school	1	1.57	(1.23–2.00)	
High school or over	1	1.69	(1.42–2.02)	
**Marriage status**				0.010
Yes	1	1.71	(1.48–1.98)	
No	1	3.11	(1.11–8.70)	
**Smoking status**				0.847
Yes	1	1.31	(0.85–2.02)	
No	1	1.76	(1.51–2.06)	
**Physical activity**				0.162
Yes	1	1.71	(1.33–2.20)	
No	1	1.66	(1.39–1.98)	
**Problem drinking**				0.126
Yes	1	2.06	(1.28–3.32)	
No	1	1.68	(1.45–1.95)	

Abbreviations: CKD, chronic kidney disease; OR, odd ratio; CI, confidence interval

* ORs were adjusted for other covariates, respectively

**Table 4:** Multinomial analysis results with the dependent variable further divided into four categories of employment status

Models		Type of employment ^d^						
		Permanent	Precarious		Self-employment		Unemployment	
	CKD	OR	OR	95% CI	OR	95% CI	OR	95% CI
**Model 1**								
	No	1						
	Yes		1.29	(0.92–1.88)	1.3	(0.90–1.88)	2.1	(1.51–2.9)
**Model 2** ^**a**^								
	No (≥ 60)	1						
	45–60		1.44	(0.98–2.11)	1.51	(1.00–2.27)	2.22	(1.52–3.2)
	< 45		0.89	(0.47–1.70)	0.77	(0.37–1.60)	1.73	(0.93–3.2)
**Model 3** ^**b**^								
	No (≥ 60)	1						
	30–60		1.48	(1.03–2.13)	1.48	(0.99–2.20)	2.34	(1.63–3.3)
	< 30		0.41	(0.15–1.08)	0.52	(0.19–1.38)	1.11	(0.46–2.6)
**Model 4** ^**c**^								
	No (≥ 60)	1						
	45–60		1.44	(0.98–2.11)	1.51	(1.00–2.27)	2.22	(1.52–3.2)
	30–45		1.79	(0.60–5.31)	1.36	(0.41–4.50)	3.12	(1.09–8.9)
	15–30		0.4	(0.10–1.53)	0.77	(0.22–2.70)	0.82	(0.25–2.7)
	< 15		0.4	(0.13–1.26)	**-**	-	2.07	(0.75–5.7)

Abbreviations: CKD, chronic kidney disease; OR odd ratio

Each model was adjusted for age, sex, marriage status, education status, household income, physical activity, smoking and drinking status, obesity, hypertension, diabetes mellitus

^a^ Estimated glomerular filtration rates of 60 or higher was classified as non-CKD, 45 to 60 and less than 45 mL/min/1.73m^2^ were classified as grades 1 and 2, respectively

^b^ Estimated glomerular filtration rates of 60 or higher was classified as non-CKD, 30 to 60 and less than 30 mL/min/1.73m^2^ were classified as grades 1 and 2, respectively

^c^ Estimated glomerular filtration rates of 60 or higher was classified as non-CKD, 45 to 60, 30 to 45, 15 to 30 and less than 15 mL/min/1.73m^2^ were classified as grades 1 through 4, respectively

^d^ Type of occupation (dependent variable) was categorized into four type of work status: full-time permanent job, self-employed, precarious job, unemployed

**Table 5:** Multinomial analysis results with the dependent variable further divided into three types of occupation

	Type of occupation						
	Unemployment (N = 14,104, 38.4%)	White collar (N = 8,746, 23.8%)		Pink collar (N = 4,552, 12.4%)		Blue collar (N = 9,310, 25.4%)	
CKD	OR	OR	95% CI	OR	95% CI	OR	95% CI
No	1						
Yes	1	0.71	(0.54–0.93)	0.64	(0.49–0.84)	0.53	(0.45–0.63)

Abbreviations: CKD, chronic kidney disease; OR odd ratio

Each model was adjusted for age, sex, marriage status, education status, household income, physical activity, smoking and drinking status, obesity, hypertension, diabetes mellitus

Type of occupation (dependent variable) was categorized into three types of occupation: white collar, pink collar, blue collar

The results of the subgroup analysis are presented in Table 3, with the analyses stratified by the independent variables. The association between CKD and employment was more prominent in the younger age group than in the older age group (OR, 2.16; 95% CI, 1.75 to 2.66). Additionally, unmarried healthy participants exhibited a significantly higher risk of unemployment than CKD participants (OR, 3.11; 95% CI, 1.11 to 8.70).

Table 4 presents the results of the multinomial analysis, with the dependent variable further categorized into four work status categories and several models representing different CKD severities. After adjusting for the potential covariates, the overall trends listed in Table 2 remained consistent. In Model 1, compared to individuals with full-time permanent jobs, those with CKD had a higher likelihood of having a precarious job (OR, 1.29; 95% CI, 0.92 to 1.88), being self-employed (OR, 1.3; 95% CI, 0.90 to 1.88), and being unemployed (OR, 2.10; 95% CI, 1.51 to 2.92). Similar trends were observed in Models 2, 3, and 4, although some components did not show significant associations with CKD severity.

Table 5 presents the results of the multinomial analysis, with the dependent variable further categorized into four types of occupation (White collar, Pink collar, Blue collar, unemployment) and CKD prevalence. After adjusting for the potential covariates, compared to individuals with unemployment, those with CKD had a lower likelihood of having a job. In other words, we can observe that the likelihood of CKD patients being unemployed is the highest, and as we move from white-collar (OR, 0.71; 95% CI, 0.54 to 0.93) to pink-collar (OR, 0.64; 95% CI, 0.49 to 0.84) to blue-collar occupations (OR, 0.53; 95% CI, 0.45 to 0.63), the odds of being employed are reduced.

## Discussion

In this study, we found a significant association between CKD and a higher likelihood of unemployment compared to that in individuals without CKD. This association was more prominent in patients with severe CKD.

Our findings are consistent with those of a previous study conducted in the United States, which identified a comparable association between renal dysfunction and labor force nonparticipation (OR, 7.94; CI, 1.60 to 39.43) [16]. The significance of our results is further emphasized by the fact that this association between CKD prevalence and unemployment was observed in Korea, a country known for its long working hours (with an average of 1993 h annually, compared with the OECD average of 1734 h in 2018) [37]. This indicates that CKD substantially hinders individuals from sustaining employment. Additionally, our study revealed that a higher proportion of patients with CKD were aged ≥ 70 years. This finding aligns with those of previous cohort studies, which have shown an age-related increase in the number of patients with CKD as the GFR declines, particularly among individuals aged ≥ 70 years [38]. This observation highlights the growing prevalence of CKD with an increasing life expectancy [1]. Considering the increasing global prevalence of CKD, these findings raise concerns regarding important social issues.

The results of the present study highlight the challenges faced by individuals with CKD in maintaining employment compared to those without CKD. However, our study had limitations in categorizing patients with CKD into specific subgroups, such as patients undergoing dialysis or kidney transplantation, due to the constraints of the questionnaire design. Despite this, we investigated the association between patients with CKD and their employment status by stratifying them based on the severity of CKD, which was determined by eGFR levels derived from serum creatinine levels. Our findings revealed a gradual increase in the risk of unemployment with an increase in CKD severity. Particularly, individuals with the most severe form of CKD experienced a substantial increase in the risk of unemployment, although the number of participants in this specific subgroup was limited. Moreover, our study is significant because in contrast with previous research, which primarily focused on comparing job retention difficulties between patients undergoing dialysis and kidney transplant [13–15], we examined the association between CKD and employment status between patients with CKD and individuals without CKD.

A previous study used data from the Third National Health and Nutrition Examination Survey conducted in the United States [16]. However, in addition to utilizing nationwide data, our study expanded the investigation by categorizing the occupational types of individuals with CKD. We discovered that the likelihood of precarious employment was higher among patients with CKD than that in individuals without CKD. Furthermore, patients with CKD displayed a notable inclination towards precarious job arrangements, self-employment, and unemployment in contrast to participants without CKD, who predominantly held permanent positions. Remarkably, this occupational pattern became more pronounced as severity of CKD, based on eGFR levels, increased. Consequently, several factors, including health-related issues, personal circumstances, and environmental factors contribute to the challenges experienced by patients with CKD in maintaining or obtaining stable employment. Patients with CKD encounter limitations in their working hours owing to regular hospital visits for dialysis treatment and heightened levels of fatigue, which adversely affects their work performance [12]. Moreover, the tendency for precarious employment among patients with CKD is supported by a significant number of individuals within this group being unemployed, which is evident from the elevated risk of unemployment observed in the subgroup with the greatest CKD severity. This outcome parallels previous findings indicating that patients in Stage 5, representing the most advanced stage of CKD, tend to quit the workforce [23]. These findings indicate that when the severity of CKD increases beyond a certain threshold, there is a tendency to quit employment.

Depending on the severity of CKD, patient’s employment status may depend on the physical intensity of the occupation. Therefore, including categorized into four types of occupation (White collar, Pink collar, Blue collar, unemployment), we can observe that the most of the CKD patients were being unemployed. The tendency to maintain employment decreased as the amount of physical labor increased. This is presumed to be due to barriers to maintaining employment caused by physical symptoms such as fatigue reported by CKD patients in previous qualitative studies [12]. Furthermore, these results provide an important implication that CKD patients working in blue collar are more easily exposed to leaving their jobs.

Our study had several limitations. First, the cross-sectional design employed in this study implies that the data were collected at a single time point. This limited our ability to establish clear causal associations, which is why the present results indicate associations rather than definitive causal relationships. Reverse causality is a drawback of cross-sectional studies. However, it can be argued that the prevalence of CKD is more likely to be a causal factor influencing the work status of patients with CKD, rather than the other way around.

Second, the survey used in this study did not consider the participants’ motivation to seek employment. Therefore, we could only examine the prevalence of CKD and absence of employment, without being able to determine whether unemployed individuals lack the drive to seek work. Further studies are required to address this issue.

Third, the physical and psychological symptoms of CKD patients were not taken into consideration. CKD patients experience various symptoms, and that the severity of these symptoms can lead to differences in their employment status. However, the survey data we used did not include questions about this information, we were unable to differentiate CKD patients based on their symptoms. Therefore, future research should be conducted based on this data to perform more detailed studies.

Finally, Due to the limitations of the data, we used, people who did not have laboratory data measured were excluded from the study. To overcome these limitations, future studies should use larger data sets to include people without laboratory data or conduct an analysis to understand their characteristics.

Despite these limitations, this study had several strengths. To our knowledge, this is the first quantitative investigation to establish an association between CKD and employment status in East Asia. Moreover, our use of KNHANES data, which accurately represent the entire Korean population, allows the extrapolation of the present findings to the broader Korean population. The reliability of the participants’ CKD and diabetes statuses was strengthened by meticulous analysis of laboratory data, including eGFR and HbA1c levels. Consequently, the outcomes of this study can potentially inform policy discussions, help integrate treatment and employment services, and thereby enhance the patients’ quality of life.

In conclusion, our study demonstrated a significant association between CKD and employment status, highlighting the impact of CKD on the prevalence of unemployment. Additionally, our findings suggest that patients with CKD have a higher likelihood of securing precarious employment than that in individuals without CKD. Patients with early- or late-stage CKD face challenges in maintaining a balance between work and health and may receive inadequate support at their workplace. Given these findings, it is crucial for clinicians and policymakers to engage in effective communication regarding the work situation of patients with CKD and address potential issues affecting their overall well-being. Active interventions, legislation, and supportive measures are necessary to improve the quality of life of patients with CKD in relation to employment.

## Data Availability

Data used in this study was from 2014 to 2021 KNHANES and can be downloaded from the KNHANES official website (https://knhanes.kdca.go.kr/).
